# Therapeutic potential of ghrelin/GOAT/GHSR system in gastrointestinal disorders

**DOI:** 10.3389/fnut.2024.1422431

**Published:** 2024-08-23

**Authors:** Yunxiao Ma, Qihui Yan, Ping Wang, Weiying Guo, Lu Yu

**Affiliations:** ^1^Department of Endocrinology and Metabolism of First Hospital of Jilin University, State Key Laboratory for Diagnosis and Treatment of Severe Zoonotic Infectious Diseases, Key Laboratory for Zoonosis Research, Ministry of Education, Institute of Zoonosis, College of Veterinary Medicine, Jilin University, Changchun, China; ^2^Department of Otolaryngology-Head and Neck Surgery of First Hospital of Jilin University, Jilin University, Changchun, China

**Keywords:** ghrelin, GOAT, GHSR, therapy, gastrointestinal disorders

## Abstract

Ghrelin, a peptide primarily secreted in the stomach, acts via the growth hormone secretagogue receptor (GHSR). It regulates several physiological processes, such as feeding behavior, energy homeostasis, glucose and lipid metabolism, cardiovascular function, bone formation, stress response, and learning. GHSR exhibits significant expression within the central nervous system. However, numerous murine studies indicate that ghrelin is limited in its ability to enter the brain from the bloodstream and is primarily confined to specific regions, such as arcuate nucleus (ARC) and median eminence (ME). Nevertheless, the central ghrelin system plays an essential role in regulating feeding behavior. Furthermore, the role of vagal afferent fibers in regulating the functions of ghrelin remains a major topic of discussion among researchers. In recent times, numerous studies have elucidated the substantial therapeutic potential of ghrelin in most gastrointestinal (GI) diseases. This has led to the development of numerous pharmaceutical agents that target the ghrelin system, some of which are currently under examination in clinical trials. Furthermore, ghrelin is speculated to serve as a promising biomarker for GI tumors, which indicates its potential use in tumor grade and stage evaluation. This review presents a summary of recent findings in research conducted on both animals and humans, highlighting the therapeutic properties of ghrelin system in GI disorders.

## 1 Introduction

Gastrointestinal (GI) disorders are extremely common worldwide and severely affect human health. They primarily comprise chronic gastritis, peptic ulcer, inflammatory bowel disease (IBD), functional disorders, and gastrointestinal tumors (1). At present, conventional treatment methods yield dissatisfactory results and lack adequate effectiveness in alleviating symptoms associated with these diseases. Hence, new GI disorder therapeutics are needed. The brain-gut hormones are widely known to influence gut motility. Various important gut hormones, including motilin, peptide YY (PYY), cholecystokinin (CCK), glucagon-like peptide-1 (GLP-1), glucose-dependent insulinotropic peptide (GIP), and ghrelin, have been identified. The use of pharmacological therapy targeting these hormones is considered a novel approach for treating GI disorders (2). Particularly, ghrelin has attracted considerable attention as a potential therapeutic target because of its diverse bioactivities.

Ghrelin is a 28-amino-acid peptide gastrointestinal hormone that was discovered by Kojima et al. in 1999 (3). It has multiple physiological functions, including the regulation of growth hormone release, energy homeostasis, glucose and lipid metabolism, cardiovascular activity, and food intake and the stimulation of gastric acid production, motility, emptying, fertility, memory, stress response, reward-seeking behaviors, and learning (4–7). To date, ghrelin is the sole gastrointestinal hormone known to exhibit orexigenic function (8). The levels of ghrelin in circulation exhibit are widely acknowledged to exhibit a strong correlation with eating habits, characterized by an elevation in ghrelin levels before meals and during periods of fasting, followed by a decrease in response to food consumption (9, 10). In addition to its function in increasing food intake, ghrelin also facilitates carbohydrate oxidation, while suppressing fat utilization, thereby promoting a state of positive energy balance (11). The orexigenic and prokinetic abilities of ghrelin make it a promising candidate for therapeutic interventions in GI disorders. Clinical trials have been conducted to assess the efficacy of ghrelin as a novel therapeutic target in various disorders such as anorexia, cachexia, functional gastrointestinal disorders, gastroparesis, and gastrointestinal cancers (12–16). The prokinetic effects of ghrelin on the GI system *in vivo* have been observed using different methods of administration in models involving surgery, opioid-induced conditions, and diabetes (17). Several animal models have also been used to explore ghrelin’s impact on migratory motor complexes (MMCs), which indicates its ability to stimulate phase II of the MMC through the vagus nerve (18, 19). Additionally, ghrelin or ghrelin agonist treatment was shown to enhance delayed gastric emptying and reduce the antral motility of mice subjected to restraint stress (20).

In recent years, the development of synthetic ghrelin agonists as potential prokinetic agents for the management of GI motility disorders, including post-operative ileus and gastroparesis, has received increasing interest. Pirnik et al. conducted a study wherein they demonstrated that the subcutaneous treatment of a ghrelin receptor agonist, specifically Dpr-(N-octanyl)-3-ghrelin, aided the stimulation of food intake through the induction of Fos expression and activation of tyrosine hydroxylase neurons in the hypothalamic arcuate nucleus (17). Additionally, the intraperitoneal administration of the ghrelin-O-acyltransferase (GOAT) inhibitor led to a decrease in food consumption in Sprague–Dawley rats. This anorexigenic effect primarily resulted from a decline in the frequency of meals, even as the size of individual meals remained unchanged compared to that in the control group (21).

This review provides a comprehensive analysis of the association between ghrelin and several significant GI diseases, as reported in recent years. The findings indicate that ghrelin exerts a defensive impact in esophageal disorders, gastric disorders, GI functional disorders, and cancer cachexia. Moreover, ghrelin exhibits promising therapeutic potential and has aided the development of numerous drugs targeting the ghrelin system, some of which are currently undergoing clinical trials. However, the research community has been divided on the involvement of ghrelin in the pathogenesis of IBD. This necessitates further investigations to ascertain the precise impact of ghrelin on IBD. Furthermore, ghrelin has been considered promising as a biomarker for GI malignancies based on its use as an indicator for assessing tumor grade and stage. In summary, this review aims to a point of reference for future studies on the correlation between ghrelin system and GI diseases.

## 2 The GOAT/ghrelin/GHSR system

### 2.1 Synthesis and post-translational modification of ghrelin

**Figure 1:**
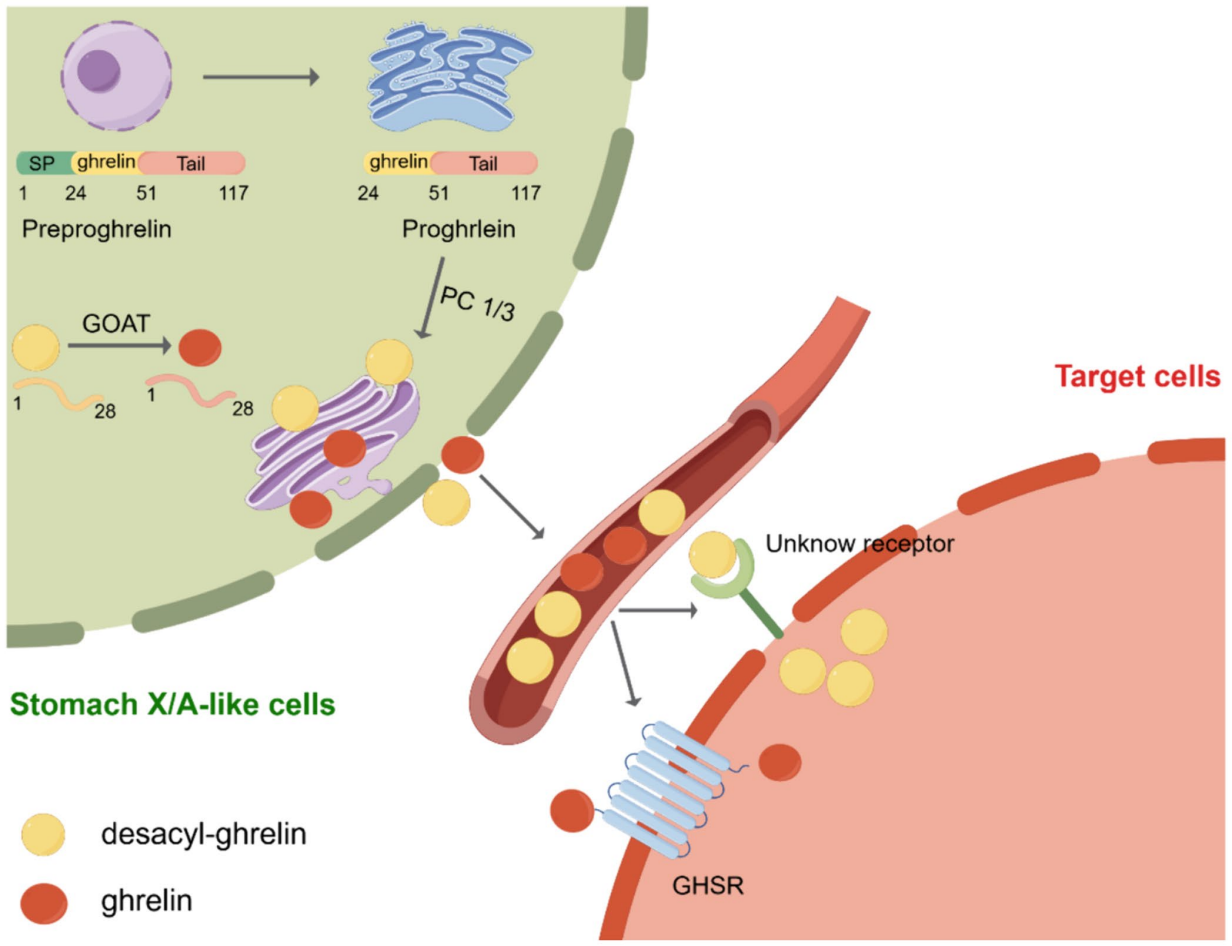
Post-translational processing of ghrelin. Initially, preproghrelin, which is composed of 117 amino acids, undergoes cleavage in the endoplasmic reticulum, resulting in the formation of proghrelin. Proghrelin undergoes acylation through the action of the enzyme known as ghrelin O-acyltransferase (GOAT). Following this, proghrelin peptides undergo additional conversion by the prohormone convertase, PC1/3, leading to the production of mature variations known as ghrelin and desacyl-ghrelin. These mature forms are then transported to the secretory vesicles located in the Golgi apparatus. After being released into the bloodstream, ghrelin attaches to the growth hormone secretagogue receptor (GHSR), initiating downstream signaling pathways. Conversely, DAG desacyl-ghrelin can also attach to its specific receptor. SP, signal peptide; GOAT, enzyme ghrelin O-acyltransferase; GHSR, growth hormone secretagogue receptor. Created by Figdraw.com.

Ghrelin was initially discovered by Kojima et al. It was known as the endogenous ligand for the growth hormone secretagogue receptor (GHSR). X/A-like cells in the stomach are the primary location for ghrelin synthesis (3). Subsequently, in 2000, Tschöp et al. demonstrated that ghrelin stimulates food consumption and influences body weight (22). Consequently, ghrelin was designated as the “hunger hormone.” Currently, ghrelin is recognized as a hormone associated with hunger. The ghrelin gene encodes a pre-proghrelin peptide composed of 117 amino acids, which undergoes several processing steps to form a mature and active peptide (23). Initially, pre-proghrelin undergoes cleavage to generate proghrelin, which is then cleaved at the C-terminal by the enzyme prohormone convertase 1/3 (PC1/3) to yield fully mature ghrelin (24). In addition, the third serine residue of ghrelin can undergo acylation through the catalytic action of GOAT (Figure 1). To remove ambiguity in the terminology of the ghrelin system, Perelló et al. conducted a survey and recommended the use of specific designations. They suggested “ghrelin” for the octanoyl-modified peptide, “desacyl-ghrelin” for the non-acylated version, “GHSR” for the ghrelin receptor and liver-expressed antimicrobial peptide 2 (LEAP2), and “LEAP2” for the newly identified endogenous GHSR antagonist/inverse agonist (25). As mentioned above, in this manuscript, we used the current consensus nomenclature for the ghrelin system.

GOAT was first discovered in 2008 by two independent laboratories (26, 27). GOAT is a transmembrane protein belonging to the family of membrane-bound O-acyltransferases (MBOAT) (28). GOAT is found not only in the stomach, brain, and pancreas but also in the intestine, ovary, serum, placenta, muscle, heart, and adrenal glands. Its distribution in various tissues is of particular importance as all of these tissues collectively contribute to food consumption control and energy balance maintenance (29–31). However, the structure of GOAT is yet to be determined, and the active site and substrate-binding sites of the enzyme remain unidentified (32). Nevertheless, through selective permeabilization experiments, the membrane topologies of GOAT, hedgehog acyltransferase (HHAT), and various lipid- and small-molecule-acylating MBOAT enzymes have been established (32–34). These investigations have shown that MBOAT enzymes exhibit intricate topological characteristics, including the presence of numerous transmembrane helices. For instance, GOAT contains 11 transmembrane domains (32). Furthermore, these aforementioned studies have demonstrated that ghrelin serves as an exclusive substrate for GOAT in the human proteome (35, 36). Therefore, GOAT inhibitors are considered a potential agent for suppressing the effects of ghrelin, including the modulation of insulin release, reduction of food intake, and attenuation of adiposity (37). In recent times, numerous GOAT inhibitors have been formulated in both academic and industrial settings. Notably, GO-CoA-Tat, a bisubstrate analog inhibitor of GOAT, is the most robust agent supporting the potential of GOAT inhibition as a therapeutic strategy for regulating ghrelin-dependent physiological processes (32, 35, 36).

Approximately 90% of the total ghrelin present in the bloodstream exists as desacyl-ghrelin, whereas less than 10% exists as ghrelin (23, 38). Initially, desacyl-ghrelin was regarded as an inactive precursor to acyl-ghrelin. Currently, limited data are available on the biological effects of desacyl-ghrelin. However, increasing evidence suggests that desacyl-ghrelin can independently or antagonistically modulate the metabolic activities of the ghrelin system, potentially through GHSR-independent pathways and the activation of an unidentified receptor (39). For instance, the induction of genome-wide alterations in gene expression related to glucose and lipid metabolism in adipose tissues, skeletal muscles, and hepatic tissues of GHSR^−/−^ mice by desacyl-ghrelin serves as substantiation for the presence of an unidentified desacyl-ghrelin receptor (40). Simultaneously, desacyl-ghrelin inhibited neuronal activity induced by ghrelin in the brainstem and hindered ghrelin/GHSR-mediated augmentation in food intake (41). Furthermore, ghrelin was previously shown to stimulate adult hippocampal neurogenesis and improve pattern separation memory (42). Jeffrey et al. used rodent models *in vitro* and *in vivo*, besides analyzing human plasma, to demonstrate that desacyl-ghrelin impairs neurogenesis and that the ghrelin: desacyl-ghrelin ratio in circulation is diminished in Parkinson’s dementia (43).

GHSR, a G-protein-coupled receptor, relays signals using a Gq/11 alpha-subunit, which leads to the activation of phospholipase C and the synthesis of inositol triphosphate (IP3), eventually leading to the release of Ca^2+^ from the endoplasmic reticulum (44). GHSR is predominantly expressed in the pituitary gland and hypothalamus and may be present in different tissues and organs, such as the thyroid gland, pancreas, spleen, myocardium, and adrenal gland (45). According to Ge et al., LEAP2, an endogenous antagonist of GHSR found in the liver and small intestine, can suppress the stimulatory effects of ghrelin on GHSR (46). Inhibiting ghrelin receptor activation by LEAP2 suppresses the major effects of ghrelin, including food consumption, growth hormone release, and glucose levels during fasting (46). Subsequently, M’Kadmi et al. discovered that both LEAP2 and its N-terminal region act as inverse agonists of GHSR and can compete against ghrelin-induced inositol phosphate synthesis and calcium mobilization (47). GHSR is currently known to exhibit both ligand-dependent and -independent functions. Both ghrelin and LEAP2 bind to GHSR in a ligand-dependent manner. Furthermore, GHSR exhibits diverse ligand-independent functions through its constitutive activity or interaction with other G protein-coupled receptors, such as dopamine receptors (D1R and D2R), orexin receptor (OX1R), serotonin receptor (5-HT2C), melanocortin-3 receptor (MC3), somatostatin receptor (SST5), and oxytocin receptor (44, 48). Moreover, evidence from an increasing number of research studies suggests that, in addition to its effects on neuroendocrine and metabolic functions, GHSR contributes to the regulation of the mesocorticolimbic pathway and influences diverse reward-related behaviors in response to various stimuli through both ligand-dependent and -independent mechanisms (49).

### 2.2 Effects of ghrelin on gastrointestinal disorders

Ghrelin is primarily produced in the stomach, although there is ongoing debate regarding its synthesis within the brain (50–52). Therefore, it is imperative to determine whether peripheral ghrelin can activate central targets and the potential mechanisms involved. Findings from recent hypotheses suggest that ghrelin may cross the blood–brain barrier (BBB), diffuse through fenestrated capillaries located in circumventricular organs (CVOs), or traverse the blood-cerebrospinal fluid (CSF) barrier (53). Banks et al. conducted experiments to assess the ability of three radiolabeled ghrelin peptides, derived from human ghrelin, mouse ghrelin, and mouse desacyl-ghrelin, to traverse BBB bidirectionally in mice (54). Mouse ghrelin exhibited saturable transport exclusively from the brain into the bloodstream, but not in the reverse direction, whereas mouse desacyl-ghrelin demonstrated non-saturable diffusion from the blood into the brain, but not vice versa. Interestingly, human ghrelin, which has two amino acids different from mouse ghrelin, can traverse the mouse BBB bidirectionally using saturable transport mechanisms (54). CVOs are regions of the brain characterized by high vascularity and a compromised BBB, exemplified by structures like median eminence (ME) and area postrema (AP). Perelló et al. observed the effects of peripheral ghrelin on specific brain regions using fluorescent ghrelin tracers. They detected a notable fluorescent signal exclusively in the arcuate nucleus (ARC) and ME in mice that received peripheral administration of fluorescein-ghrelin at a low dose (55). However, in mice that received peripheral administration of a high dose of fluorescein-ghrelin, the fluorescein signal was observed not only in the ARC and ME but also in the AP and paraventricular nucleus (PVN), suggesting that circulating ghrelin can traverse fenestrated capillaries and reach certain CVOs (55). In addition, subsequent investigation illustrated that plasma fluorescent-ghrelin exhibits selective internalization by cells forming the CSF barrier, including ependymal cells of the choroid plexus and β-type tanycytes in mice. This internalization enables the transport of ghrelin to the CSF, promoting diffusion to the periventricular hypothalamic regions and subsequently mediating diverse effects (56).

However, despite current evidence suggesting limited peripheral-to-central nervous system (CNS) passage of ghrelin, the central ghrelin system plays a crucial role in the regulation of feeding behaviors. Accordingly, significant GHSR expression is observed in the brain areas that regulate food intake, such as the hypothalamus, brainstem, hippocampus, amygdala, and ventral tegmental area (VTA) (57, 58). Furthermore, microinjection of ghrelin into regions of the brain abundant in GHSRs has been shown to motivate behaviors aimed at acquiring and consuming food (55). In the ARC, GHSR is expressed primarily in neuropeptide Y/agouti-related peptide (NPY/AgRP) neurons and proopiomelanocortin/cocaine and amphetamine-regulated transcript (POMC/CART) neurons, but to a lesser degree. (59–61). NPY/AgRP neurons play a role in appetite enhancement. The stimulation of POMC/CART neurons promotes satiety (62). Interestingly, ghrelin stimulates NPY and AGRP orexigenic peptide transcription, but not that of the anorectic peptide POMC (63). In addition, mice with ablated ARC do not exhibit increased appetite when subcutaneously administered ghrelin, whereas they exhibit orexigenic traits when ghrelin is administered centrally (55). Consequently, the ARC is determined to be a critical mediator of the acute orexigenic response to ghrelin administration. Moreover, the AP, which is situated in the caudal brainstem and is part of the dorsal vagal complex (DVC), which also includes the nucleus of the solitary tract (NTS) and the dorsal motor nucleus of the vagus (DMV), acts as a critical nucleus where peripheral ghrelin transmits orexigenic messages. Previous research has demonstrated that rats with AP lesions do not consume more food when they receive chronic peripheral ghrelin injections, in contrast to the observations in control groups (64). Also, Cabra et al. demonstrated that AP-ablated mice exhibit slower gastric emptying induced by circulating ghrelin (65). Meanwhile, ghrelin promotes food intake through the ventral tegmental area (VTA), which contains dopaminergic neurons implicated in reward-based eating behaviors (66). In contrast to sham-operated rats, rats with VTA lesion maintained regular intracerebroventricular ghrelin-driven feeding; however, they consumed and explored rewarding food less frequently (67). Similarly, intra-VTA infusions of ghrelin have been shown to stimulate food intake and increase the desire to eat palatable foods (68). Conversely, rats subjected to the intra-VTA administration of [Lys-3]-GHRP-6, an antagonist of the ghrelin receptor, exhibited selectively diminished feeding and decreased the motivation for obtaining palatable food compared to control and ghrelin-treated rats (68). Nonetheless, it is currently unknown whether peripheric ghrelin can reach the VTA directly.

**Figure 2:**
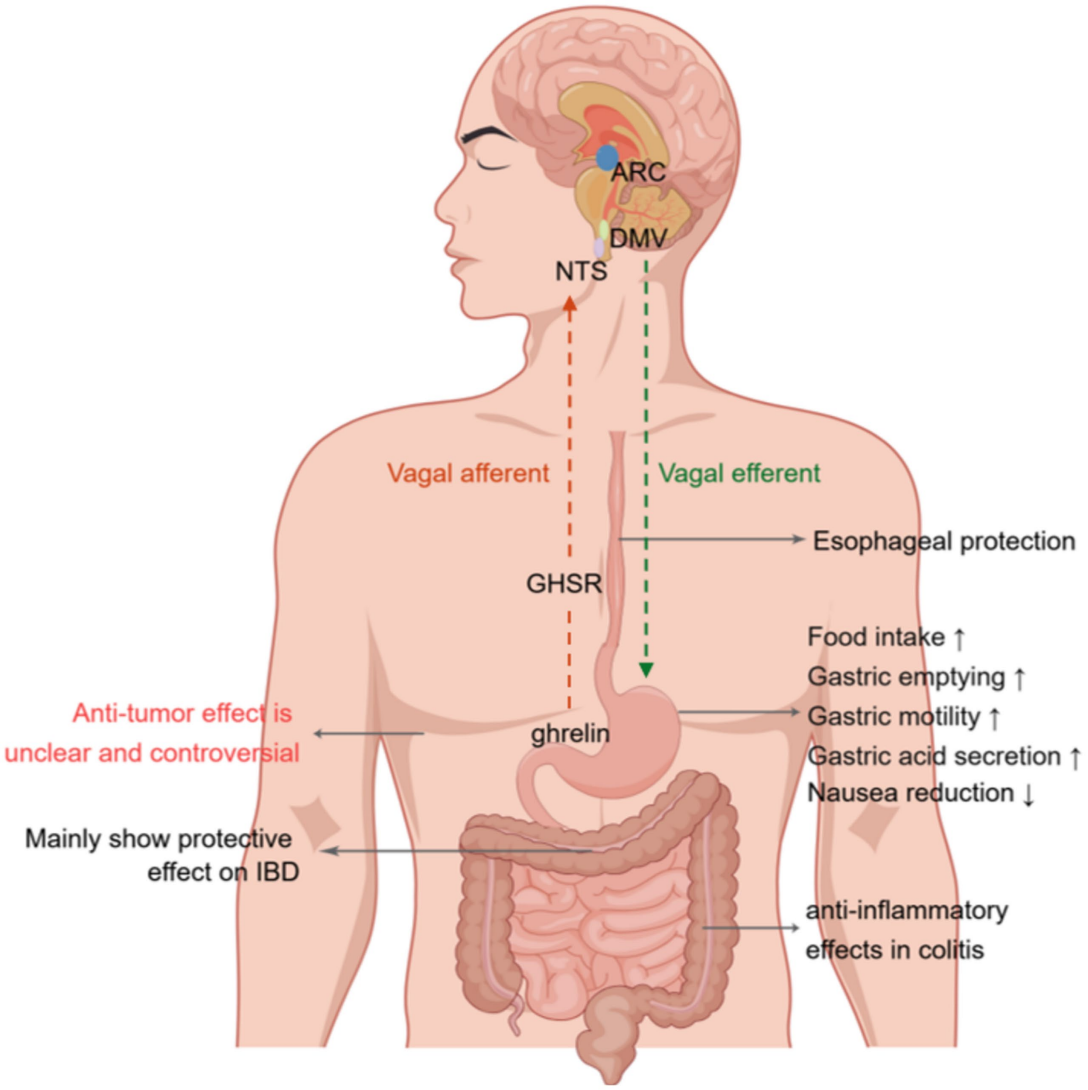
Effects of ghrelin on gastrointestinal disorders. Ghrelin binds the growth hormone secretagogue-receptor1a (GHSR) in vagal afferent nerve fibers. This binding leads to the transmission of signals to the nucleus of the solitary tract (NTS). Neuropeptide Y (NPY) neurons projecting onto the arcuate nucleus (ARC) of the hypothalamus activate the signal from the NTS. Eventually, this message is relayed to the dorsal motor nucleus of the vagus (DMV) via vagal efferent fibers, resulting in increased gastric contractions, acid production, and gastrointestinal motility. Furthermore, it affects the paraventricular nucleus (PVN) and ARC of the hypothalamus by traversing the blood–brain barrier, thereby augmenting the release of growth hormone and stimulating appetite. Additionally, it modulates the activity of neurons in the area postrema, leading to a reduction in nausea. NTS, nucleus tractus solitaries; ARC, arcuate nucleus; DMV, dorsal motor nucleus of the vagus; GHSR; growth hormone secretagogue receptor; IBD, inflammatory bowel disease. Created by Figdraw.com.

The vagus nerve facilitates bidirectional connection between the gut and brain. Specifically, vagal afferent neurons convey signals from the gut to the brain, whereas vagal efferent neurons transmit signals from the brain to the gut. However, the involvement of vagal afferent nerves in mediating the effects of ghrelin remains a contentious topic (69) (Figure 2). Research shows that ghrelin binds to GHSR in vagal afferent neurons within the GI tract. These neurons relay mechanical, osmosensory, and chemosensory signals to the NTS located in the brainstem. The NTS is an area of the brain associated with visceral reflexes and establishes connections with the hypothalamus to regulate feeding (70, 71). This neural communication eventually excites the preganglionic motor neurons of the DMV, which subsequently activate postganglionic cholinergic neurons, leading to enhanced GI motility (70, 71). Notably, surgically removing and selectively destroying the vagal afferent nerves can completely suppress the orexigenic effects of peripheral ghrelin, while vagotomized rodents can retain their responsiveness to centrally injected ghrelin (72, 73). Conversely, it has been suggested that peripheral ghrelin does not need vagal afferent nerves to stimulate food intake (74). Arnold et al. used the subdiaphragmatic vagal deafferentation method to demonstrate that vagotomized rats consume more food than sham-lesioned rats after receiving peripheral ghrelin injections (74). This finding indicates that the activation of neural orexigenic pathways in response to peripheral ghrelin is not strictly dependent on vagal afferent nerves (74). Consequently, the precise role played by the vagus nerve in ghrelin’s orexigenic effects warrants further investigation to elucidate this role.

## 3 Ghrelin system and esophageal disorders

The impact of ghrelin treatment on the recovery process in esophageal injury remains uncertain. Previous clinical and experimental investigations have shown the levels of ghrelin in Barrett’s esophagus and gastroesophageal reflux disease (GERD), although the findings were inconsistent (75–77). Thomas et al. conducted a case–control study investigating the correlation between the incidence of Barrett’s esophagus and serum ghrelin levels (78) (Table 1). Their findings indicated that an elevated ghrelin level is linked to a greater likelihood of Barrett’s esophagus than that in the control group. Meanwhile, ghrelin concentration did not appear to be correlated to the frequency of GERD symptoms. Additionally, a separate clinical study revealed a favorable connection between serum ghrelin levels and the incidence of Barrett’s esophagus, but also showed an inverse association between serum ghrelin levels and GERD (75). Conversely, GERD rats exhibit elevated levels of plasma ghrelin, and ghrelin signaling in these rats may be inhibited owing to the reduced synthesis of melanin-concentrating hormone (MCH) and orexin in the hypothalamus (94). Findings from another animal study showed that impaired ghrelin signaling may be implicated in GI dysmotility in GERD rats. Furthermore, the administration of rikkunshito promoted a diminished response to ghrelin, thereby improving gastrointestinal motility. Consequently, enhancing ghrelin signaling may be a novel method for treating GERD.

**Table 1:** Circulating ghrelin levels in gastrointestinal disorders patients.

Study groups	Study methods	Level of Ghrelin	Correlations with clinical parameters	Reference
*N* = 320 BE; *N* = 316 GERD; *N* = 317 control	a case–control study	↑ BE vs. GERD and control	higher levels correlation with an increased risk of BE	(78)
*N* = 261 GNCA; *N* = 98 EGJA; *N* = 441 control	a case–control study	↓ GNCA vs. control; ↓ EGJA vs. control	lower levels correlation with an increased risk of GNCA and EGJA	(79)
*N* = 16 *H. pylori* positive; *N* = 14 *H. pylori* negative	a case–control study	↓ *H. pylori* positive vs. *H. pylori* negative	lower levels correlation with an increased risk of *H. pylori* infection	(80)
*N* = 82 *H. pylori* positive; *N*= 70 *H. pylori negative*	a cross-sectional study	↓ *H. pylori* positive vs. *H. pylori* negative	lower levels correlation with persistent *H. pylori* infection and the severity of gastric pathology of the corpus in dyspeptic patients	(81)
*N* = 22 AIG with delayed GE; *N* = 19 AIG with normal GE	a case–control study	↓ AIG with delayed GE vs. AIG with normal GE	lower levels correlation with an increased risk of delayed GE in AIG	(82)
*N* = 53 UC (27 active, 26 inactive); *N* = 43 Crohn’s disease; (15 active, 28 inactive); *N* = 40 control	a case–control study	↑ active IBD vs. inactive IBD and control	ghrelin secretion increases in active IBD	(83)
*N* = 42 FD; *N* = 14 control	a case–control study	↓ FD vs. control	lower levels correlation with FD	(84)
*N* = 36 EPS; *N* = 76 PDS; *N* = 39 NERD; *N* = 20 control	a case–control study	↓ PDS and NERD vs. control	lower levels correlation with the pathophysiology of PDS through its effect on GE	(85)
*N* = 16 IBS-D; *N* = 16 IBS-C; *N* = 16 control	a case–control study	↑ IBS-D vs. IBS-C and control	ghrelin may play a vital role in IBS pathophysiology	(86)
*N* = 220 gastroesophageal cancers; *N* = 125 control	a case–control study	↓ gastroesophageal cancers vs. control	serum ghrelin is inversely associated with gastric cancer; lower serum ghrelin was also associated with ESCC	(87)
*N* = 82 OSCC; *N* = 82 control	a case–control study	↓ OSCC vs. control	lower levels correlation with an increased risk of OSCC	(88)
*N* = 298 ESCC; *N* = 518 GCA; *N* = 258 GNCA; *N* = 770 control	a prospective cohort study	↓ GNCA and GCA vs. control; ↑ ESCC vs. control	lower levels correlation with an increased risk of GNCA and GCA; but correlation with a reduced risk of GCA	(89)
*N* = 284 colon cancers; *N* = 239 rectal cancers; *N* = 523 control	a case–control study	↓ colon and rectal cancers vs. control	lower levels correlation with an increased risk of colon and rectal cancers within 10 years of blood draw; but with a decreased risk of colorectal cancer more than 20 years after blood draw	(90)
*N* = 21 cancer cachexia; *N* = 24 cancer but without cachexia; *N* = 23 control	a case–control study	↑ cancer cachexia and cancer but without cachexia vs. control	higher levels correlation with an increased risk of cancer cachexia	(91)
*N* = 43 colorectal adenomas with high-grade dysplasia; *N* = 49 colorectal adenomas with low-grade dysplasia	a case–control study	↑ high-grade adenoma vs. low-grade adenomas	higher levels correlation with an increased risk of dysplasia in colorectal adenomas	(92)
*N* = 95 colon cancer; *N* = 39 control	a case–control study	↑ colon cancer vs. control	higher levels correlation with an increased risk of colon cancer	(93)

↓↑, decrease/increase level; BE, Barrett’s esophagus; GERD, gastroesophageal reflux disease; GNCA, gastric noncardia adenocarcinoma; EGJA, esophagogastric junctional adenocarcinoma; AIG, autoimmune gastritis; GE, gastric emptying; UC, ulcerative colitis; IBD, inflammatory bowel disease; FD, functional dyspepsia; EPS, epigastric pain syndrome; PDS, postprandial distress syndrome; NERD, non-erosive reflux disease; IBS-D, irritable bowel syndrome diarrhea; IBS-C, irritable bowel syndrome; ESCC, esophageal squamous cell carcinoma; OSCC, oesophageal squamous cell carcinoma; GCA, gastric cardia adenocarcinoma; EGJA, esophagogastric junctional adenocarcinoma.

In addition, individuals with low initial serum ghrelin levels are more susceptible to the onset of esophagogastric junctional and gastric adenocarcinomas, indicating the potential involvement of ghrelin in the development of these cancers (79). Nevertheless, the introduction of ghrelin does not affect the apoptosis of the OE-19 Barrett adenocarcinoma cell line when tested *in vitro*. Conversely, treatment with ghrelin appears to hinder the progression of Barrett’s carcinogenesis by suppressing the expression of proinflammatory factors (95). Based on findings reported to date, additional research is necessary to clarify the underlying mechanism.

## 4 Ghrelin system and gastric disorders

The stimulatory effects of ghrelin on gastric motility and gastric emptying have been widely acknowledged (96). Research has demonstrated that the peripheral application of ghrelin leads to an increase in gastric acid secretion in a dose-dependent manner, which is mediated by the activity of the vagal nerve and the release of histamine (97). Conversely, the central administration of ghrelin elicits an inhibitory response, suppressing gastric acid release (98). The gastroprotective effects of ghrelin have been demonstrated in various experimental models of gastric ulcers. Studies have shown that both central and peripheral administration of ghrelin effectively prevents the formation of gastric ulcers caused by ethanol in rats (99) (Table 2). Additionally, pretreatment with ghrelin was shown to inhibit the formation of stomach ulcers induced by immersion in water and restraining stress, gastric ischemia/reperfusion, intragastric concentrated hydrochloric acid administration, or alendronate treatment (100, 107).

**Table 2:** Effects of the ghrlein on gastrointestinal disorders in animal experiments.

Agents	Agent doses	Model of the study	Effects	Reference
Exogenous ghrelin, GHSR antagonist [D-Lys3]-GHRP-6, and rikkunshito	Single i.p. injection (1 or 3 nmol/mouse); [D-Lys3]-GHRP-6 (0.2 μmol/mouse); orally rikkunshito(100, 250 mg/kg)	Male ICR mice exposed to acute restraint stress	May be useful in the treatment of decreased gastric function caused by stress	(20)
Exogenous ghrelin	Intracerebroventricularly (4–4,000 ng/rat); s.c. injection (80 μg/kg)	The ethanol-induced gastric ulcers rat model	Dose-dependently reduced ethanol-induced gastric ulcers	(99)
Exogenous ghrelin	Single i.p. injection (40 g/kg)	A HCl-induced gastric damage rat model	Reduced (43%) the gastric lesions caused by concentrated acid	(100)
Exogenous ghrelin	i.p. injection (0.05–2.0 nmol/mouse) for 3 days	A colitis mouse model	Ameliorated the severity of colitis; abrogated body weight loss, diarrhea, inflammation; and increaed survival	(101)
Exogenous ghrelin	i.p. injection (0.05 mg/kg) for 5 days	A mild necrotizing enterocolitis newborn Sprague–Dawley rat model	Recovered the mild NEC-induced changes to the histology, HF-HRV, and myenteric phenotype in a vagally dependent manner	(102)
Exogenous ghrelin	i.p. twice a day (8 nmol/kg) for 7 days	An acetic acid-induced colitis rat model	Accelerated the healing of colitis	(103)
Exogenous ghrelin	i.p. injection (100 nmol/kg) twice daily for 10 days	DSS-induced colitis mice model	Aggravates colitis	(104)
Rikkunshito, a traditional Japanese Kampo medicine that potentiates ghrelin signaling	Oral gavage Rikkunshito twice daily at 1 g/kg/day for 7 days	A cancer cachexia rat model induced by human gastric cancer-derived 85As2 cells	Rikkunshito ameliorated cancer anorexia-cachexia symptoms may involve alleviation of ghrelin resistance via enhancement of ghrelin signaling	(153)
HM01, ghrelin receptor agonist	Oral gavage (10 mg/kg) for 14 consecutive days	A Colon-26 (C26) tumor-bearing mice model	Increased food intake, body weight, fat mass, muscle mass, and bone mineral density while it decreased energy expenditure	(105)
Exogenous ghrelin	i.p. injection (3 nmol/day) for 7 days	AOM/DSS-induced inflammation-associated colon carcinogenesis model	Suppressed inflammation-associated colorectal carcinogenesis	(106)

i.p., intraperitoneal; GHSR, growth hormone secretagogue receptor; AOM, azoxymethane; DSS, dextran sodium sulfate; HF-HRV, high frequency spectrum of heart rate variability; s.c., subcutaneous injection.

Numerous studies have investigated the concentrations of ghrelin in the bloodstream and tissues of individuals infected with *Helicobacter pylori* (*H. pylori*). Circulating ghrelin and GOAT levels are substantially lowered in *H. pylori*-infected individuals compared to that in non-infected individuals (80, 108). Moreover, the levels of circulating ghrelin were found to decrease in correlation with the degree of *H. pylori*-induced gastritis and the severity of chronic atrophic gastritis (81, 109, 110). Furthermore, ghrelin levels were found to be significantly lowered in individuals with autoimmune gastritis who exhibited delayed gastric emptying and impaired autonomic function. This indicates that ghrelin may have an important function in the delayed gastric emptying observed in these individuals (82). Nevertheless, differences in GHSR mRNA expression among the different groups were not significant, whereas GHS-R1b expression was considerably higher in patients with *H. pylori* infection and gastritis. Bahar et al. proposed that intermediaries of the ghrelin axis, such as GHS-R1b, could potentially serve as a clinical target for gastric disorders (111).

Additionally, previous research has demonstrated that ghrelin can enhance blood circulation in the digestive system in a sepsis model and potentially prevent the apoptosis of stomach mucosa cells by regulating apoptosis-related elements in gastric tissues, including B-cell lymphoma 2 (Bcl-2), Bcl-2-associated X (Bax), and caspase-3 proteins (112). Furthermore, Slomiany et al. indicated that the modulatory effects of ghrelin on gastric mucosal reactions to *H. pylori* lipopolysaccharide (LPS) rely on stimulation by phosphatidylinositol 3-kinase (PI3K), which is contingent on the PLC/PKC signaling pathway (113). Moreover, ghrelin exhibits a counteractive effect on the proinflammatory outcomes induced by *H. pylori* by interfering with the activation of AP-1 through the p38/ATF-2 pathway while concurrently enhancing Src/Akt-dependent cNOS phosphorylation (114). Collectively, ghrelin holds potential as a novel therapeutic target for managing gastric disorders.

## 5 Ghrelin system and inflammatory bowel disease

IBD is a chronic inflammatory disease affecting the digestive system. There are two types of IBD: ulcerative colitis (UC) and Crohn’s disease (CD) (115). The association between ghrelin and IBD remains ambiguous. Numerous investigations have demonstrated the favorable impact of ghrelin on individuals afflicted with IBD. In *in vitro* and *in vivo* experiments, ghrelin could protect against TNF-α-induced apoptosis caused by dextran sulfate sodium (DSS) or 2,4,6-trinitrobenzene sulfonic acid (TNBS) in Caco-2 cells, which are intestinal epithelial cells, and mouse colitis models. This protective effect was mediated via the regulation of the unfolded protein response and modulation of caspase-3, Bax, and Bcl-2 expression. However, notably, this protective effect could be disrupted by the antagonist of GHSR, [D-lys3]-GHRP-6 (116). In the present study, ghrelin treatment in a comparable animal model, either at the onset of the illness or a few days after colitis was established, induced a mitigating effect on the disease’s clinical and histopathologic severity. The therapeutic outcome was found to be linked with the inhibition of both inflammatory and Th1-induced autoimmune responses, along with an increase in IL-10 levels (101). Furthermore, previous studies have indicated that the administration of ghrelin can improve the characteristics of newborn rats suffering from mild necrotizing enterocolitis and expedite healing in rats with acetic acid-induced colitis (102, 103). Additionally, Yeon et al. demonstrated that GHSR KO mice exhibit heightened susceptibility to experimental colitis, characterized by elevated levels of proinflammatory cytokines and diminished expression of gut tight junction proteins (117).

Nevertheless, certain studies suggest that ghrelin may exert detrimental effects on IBD. Previous research has demonstrated that individuals with IBD, particularly individuals with active inflammation in UC, exhibit increased circulating levels of ghrelin (83, 104). Additionally, the administration of exogenous ghrelin has been found to worsen experimental colitis (83, 104). Moreover, the levels of circulating ghrelin in patients with UC and CD are associated with the levels of TNF-α, C-reactive protein, and fibrinogen and the erythrocyte sedimentation rate, while exhibiting a negative correlation with nutritional status parameters (118). In addition, the mRNA levels of ghrelin and its receptor were elevated in mice with TNBS-induced colitis, and ghrelin was found to enhance IL-8 promoter activity and stimulate the NF-κB/IκB pathway in a human colonic epithelial cell line (119). According to the aforementioned findings, exogenous ghrelin treatment in animal models has facilitated, in certain studies, an improvement in the disease course. However, this effect has not been observed consistently across studies. Notably, ghrelin levels are elevated in IBD. However, the clinical significance of the increase in ghrelin expression remains unclear. Some researchers speculate that the upregulation of ghrelin represents a compensatory mechanism aimed at mitigating tissue damage subsequent to intestinal inflammation, suggesting that such damage may induce the secretion of endogenous ghrelin (120). Nonetheless, the therapeutic efficacy of this elevated endogenous ghrelin concentration remains to be elucidated through further investigations on IBD.

## 6 Ghrelin system and functional gastrointestinal disorders

### 6.1 Gastroparesis

Gastroparesis is distinguished by the delayed emptying of the stomach without any mechanical obstruction (121). Diabetic, postsurgical, and idiopathic gastroparesis are the primary forms of the condition. Ghrelin strengthens gastric emptying and stimulates contractile activity in the GI tract (122). In a recent study, ghrelin was found to induce the depolarization of pacemaker potential in the interstitial cells of Cajal in a dose-dependent manner within the small intestine of mice. GHRP-6, a ghrelin receptor antagonist, completely disrupted this effect. These findings suggest that ghrelin likely modulates interstitial cells of Cajal by interacting with their receptor, leading to alterations in electrical signals across the digestive system and subsequently influencing gastrointestinal motility (123). The prokinetic effects of ghrelin should be taken into account when considering pharmacologic interventions for gastroparesis, as they can enhance gastric emptying and alleviate symptoms. However, the limited half-life and vulnerability in the bloodstream impair the efficacy of ghrelin. Consequently, the development of small molecule ghrelin receptor agonists with extended receptor activity emerges as a promising therapeutic approach for addressing gastrointestinal motility disorders.

In recent years, various ghrelin receptor agonistshave been tested in clinical trials on patients with diabetic gastroparesis. These agonists have shown the ability to enhance gastric emptying and alleviate symptoms associated with gastroparesis (124). Among these agonists, relamorelin has been extensively investigated for its potential in the treatment of gastroparesis, whereas others were not tested further owing to their limited effectiveness (125, 126). Relamorelin, an injectable agonist of the ghrelin receptor, exhibits a potency approximately six times greater than that of endogenous ghrelin, along with enhanced stability and an extended plasma circulating half-life in comparison to ghrelin. A prior investigation demonstrated that relamorelin was approximately 100 times more potent than ghrelin and effectively reversed delayed gastric emptying in a morphine-induced model of gastroparesis in Sprague–Dawley rats. Furthermore, the oral administration of relamorelin notably improved gastrointestinal transit in the small intestine (127). In addition, a recently published meta-analysis indicates that, compared to a placebo, relamorelin exhibits both effectiveness and tolerability in the treatment of diabetic gastroparesis (128).

Notably, in a double-blind phase 2 trial with 204 individuals suffering from diabetic gastroparesis, the primary endpoint was the gastric emptying half-time. Relamorelin administered twice daily enhanced gastric emptying and improved the vomiting severity score compared to that in the placebo, but it did not significantly improve other GI manifestations such as belly ache and satiety (129). Meanwhile, another study, which is the largest conducted thus far, also had a phase 2 double-blinded design and included 393 individuals with diabetes who were experiencing symptoms of gastroparesis ranging from moderate to severe (130). This investigation aimed to examine the impact of a 12-week treatment regimen using relamorelin, with the frequency of vomiting serving as the primary outcome measure. Despite the absence of improvement in the primary endpoint, noteworthy findings were reported, including a significant reduction in manifestations such as bloating, nausea, postprandial fullness, and bellyache. Additionally, all dosage groups treated with relamorelin exhibited enhanced gastric emptying compared to the placebo (130). These findings collectively suggest that relamorelin is effective in managing diabetic gastroparesis in individuals who have symptoms of active vomiting, as demonstrated in phase 2 trials, and the compound is currently being assessed in phase 3 trials (129, 130).

### 6.2 Functional dyspepsia

FD is a prevalent digestive disorder that presents with persistent epigastric discomfort, including pain, burning, and postprandial fullness, without any identifiable organic cause (131). However, despite numerous proposed pathogenic mechanisms, the exact etiology of FD remains elusive, and its pharmacological treatment is inadequately understood. The role of ghrelin in regulating gastric motility has been investigated and linked to the development of FD.

For example, the intravenous administration of ghrelin twice daily for half a month led to a notable enhancement in appetite and a potential increase in the daily food intake among patients with FD experiencing appetite loss (132). Furthermore, in a human study, rikkunshito treatment notably increased ghrelin and the ghrelin/desacyl-ghrelin ratio, whereas the levels of desacyl-ghrelin exhibited a declining pattern (133). Meanwhile, Arai et al. observed a notable amelioration of GI symptoms in people diagnosed with FD in response to treatment with the traditional Japanese herbal medicine Rikkunshito. This treatment was found to elevate plasma ghrelin levels (134). Moreover, Takamori et al. reported a significant reduction in the levels of fasting des-acyl and total ghrelin in patients with FD compared with that in controls, although the fasting and postprandial ghrelin levels in the two groups did not show statistically significant differences (84, 135). Besides, a recent study has demonstrated a notable decrease in plasma ghrelin levels among individuals diagnosed with FD in comparison to that in healthy volunteers (85). These findings substantiate the potential efficacy of ghrelin as a therapeutic intervention for FD. Nevertheless, the enduring consequences and adverse reactions associated with peptide hormone therapies remain unclear. Consequently, additional investigations elucidating the underlying mechanisms are imperative to confirm the effectiveness of ghrelin as a treatment target in FD.

### 6.3 Irritable bowel syndrome

IBS is a functional gastrointestinal disorder that presents with symptoms such as bloating, altered bowel habits, pain, and discomfort, without any identifiable physical cause (136). Distinct subtypes of IBS are categorized as diarrhea-predominant IBS (IBS-D), constipation-predominant IBS (IBS-C), and alternating-pattern IBS (137). A comprehensive understanding of ghrelin’s function in IBS pathophysiology is warranted.

Two recent retrospective studies have reported a notable rise in plasma ghrelin levels in individuals with IBS-D and heightened staining intensity in the antral mucosal gland in individuals with IBS-C compared to that in control groups (86, 138). In addition, two recent studies have indicated a reduction in the GT genotype and the T allele of the GHRL rs696217 polymorphism in patients with IBS compared to that in healthy individuals. This suggests that ghrelin’s polymorphisms are closely associated with vulnerability to IBS development and may contribute to the pathogenesis of IBS (139, 140). The phenomenon of a disrupted intestinal barrier function, commonly referred to as “leaky gut,” has been observed in various human disorders, such as IBS, IBD, and Alzheimer’s disease (141). Ishioh et al., have found that ghrelin exerts a central effect in ameliorating leaky gut by modulating adenosine A2B receptors, subsequently activating the vagal efferent pathway (142). Moreover, in a Wistar rat model of stress-induced IBS, the subcutaneous injection of ghrelin twice weekly demonstrated an antinociceptive effect by regulating TRPV1/opioid systems. However, the effect was partially inhibited by the ghrelin antagonist [D-Lys3]-GHRP-6 (143). Collectively, altered ghrelin could subsequently influence gastric motility and potentially contribute to IBS pathophysiology. Consequently, ghrelin may hold promise as a novel treatment for IBS.

## 7 Ghrelin system and gastrointestinal cancer

Gastrointestinal cancers are a significant contributor to global morbidity and mortality rates. The current literature presents inconclusive and contentious findings on the association between ghrelin and gastrointestinal cancer. Given that ghrelin synthesis predominantly occurs within the GI tract, its production may potentially be influenced by the onset and progression of cancer in this region.

### 7.1 Ghrelin system and the risk of gastrointestinal cancers

A large body of clinical evidence establishes a correlation between the serum concentration of ghrelin and the occurrence of GI cancers. Murphy et al. documented a negative association between serum ghrelin levels and the susceptibility to esophageal malignancies, particularly esophageal squamous cell carcinoma. Notably, individuals with lowered ghrelin levels exhibit a seven-fold greater likelihood of developing this specific histological subtype of GI cancer (87, 88). The association between ghrelin levels and disease susceptibility has been confirmed in a more extensive cohort study (89). While ghrelin alone may not possess sufficient utility as a biomarker for assessing the likelihood of gastric malignancies, its combination with other early detection biomarkers like pepsinogen I and pepsinogen I/II ratio can help enhance diagnostic accuracy (89).

However, contrary to the findings of Murphy et al., a notable positive association between reduced serum ghrelin level and the likelihood of colorectal cancer was observed in a comprehensive prospective case–control study conducted over a decade (90). In addition, an investigation on 295 individuals diagnosed with gastric cancer, in which data from four gene expression microarrays were used, led to the identification of 12 genes that were upregulated and 59 genes that were downregulated, with elevated GHRL expression being linked to unfavorable overall survival outcomes in patients with gastric cancer (144). Moreover, a comprehensive investigation was conducted to assess the levels of key elements of the ghrelin/GOAT/GHSR system in gastroenteropancreatic neuroendocrine tumors. The findings revealed a significant upregulation of GOAT in tumor specimens in comparison to adjacent non-tumor and normal tissues, suggesting the potential utility of GOAT as an innovative diagnostic biomarker (145). In conclusion, a greater number of clinical studies are required to establish the association between levels of ghrelin and GI cancers as well as to determine the viability of ghrelin as a GI tumor marker.

### 7.2 Ghrelin system and cancer cachexia

CC is a complex condition characterized by a disruption in the protein and energy balance, sarcopenia, abnormal metabolism, and progressive functional decline (146, 147). Over the past few years, an increasing body of research has focused on understanding the role of ghrelin in CC (148). A recent study conducted on a murine model examined the potential therapeutic effects of ghrelin and desacyl-ghrelin. Treatment with ghrelin and desacyl-ghrelin effectively mitigated cachectic symptoms, enhanced the nutritional status, impeded muscle and adipose tissue atrophy, and lowered serum TNF-α levels. Furthermore, ghrelin/desacyl-ghrelin treatment suppressed calpain activity, inhibited atrogin-1 expression, and augmented Akt activity in skeletal muscles (149). Furthermore, ghrelin inhibited cachexic muscle atrophy, which is induced as a consequence of chronic renal failure, thermal injury, cancer, and chemotherapy. This is achieved through the augmentation of muscle protein synthesis and the reduction of proteolysis (150).

In recent years, anamorelin, a particular ghrelin receptor agonist, has gained approval for use in treatments (151, 152). In accordance with findings from previous investigations, this medication showed efficacy in enhancing body weight, lean body mass, and appetite, as evidenced by findings from a randomized, double-blind study involving patients with CC (13). Nevertheless, anamorelin did not exhibit the potential to ameliorate motor function or overall survival in these individuals (13). Moreover, in mice with colon-26 tumors, the oral intake of HM01, a ghrelin receptor agonist, enhanced food consumption, increased body weight, augmented fat and muscle mass, elevated bone mineral density, and reduced energy expenditure (105). Additionally, anorexia is frequently observed in individuals with CC, even though they have high levels of ghrelin, indicating the potential development of ghrelin resistance in these patients (91). Garcia et al. speculated that ghrelin resistance observed in patients with cancer cachexia may be similar to insulin resistance in patients with type 2 diabetes mellitus, which can be mitigated by treatment with high doses of insulin (91). This may explain why patients with cancer cachexia exhibit increased levels of endogenous ghrelin without a corresponding increase in food intake, but they can show an increase in appetite and food intake when they receive exogenous ghrelin at levels three to four times higher than baseline (91). Furthermore, Terawaki et al. (2017) conducted a study using a rat model of cancer anorexia-cachexia induced using 85As2 cells derived from human gastric cancer; the authors reported ghrelin resistance in the study model. However, treatment with rikkunshito, which promotes ghrelin signaling, ameliorated symptoms related to cancer anorexia-cachexia (153). These findings highlight the potential of focusing on ghrelin as a treatment for CC, while also emphasizing the need for further research to enhance the efficacy of current pharmaceutical interventions.

### 7.3 Ghrelin system and GI cancer grade and stage

At present, numerous studies have demonstrated variations in the expression level of ghrelin across different tumor stages, suggesting its potential as a significant indicator for tumor grade or stage evaluation. A prior prospective study, conducted on 92 patients, revealed high levels of ghrelin and its receptor in colon carcinoma cells, with a decrease observed in less differentiated tumors. This finding indicates the potential importance of ghrelin in the early stages of tumorigenesis (92). Moreover, several studies have indicated a noteworthy escalation in the susceptibility to non-cardia gastric cancer and cancer at the junction between the esophagus and stomach in patients with initial lower levels of ghrelin; these alterations become apparent at an early stage of cancer development (87). Analogously, another study revealed an elevation in ghrelin levels during the later stages of the disease; this indicates a positive correlation between ghrelin levels and the degree of differentiation and is especially more pronounced in instances of inadequately differentiated colorectal cancer (93).

Furthermore, the initial identification of ghrelin expression in esophageal squamous cell carcinoma was achieved through immunohistochemistry, wherein tissue ghrelin levels exhibited significant associations with the extent of differentiation, level of tumor invasion, lymph-vascular invasion, and tumor stage (154). However, no notable association was observed between ghrelin expression levels and patient survival (154). In addition, a study conducted on live organisms indicated that ghrelin treatment effectively inhibited tumor progression in the colon of mice with inflammation-related colon cancer caused by azoxymethane/DSS (106). In summary, ghrelin holds potential in the assessment of GI tumors, although further experimentation is required to validate this assertion.

## 8 Conclusion and prospects

In conclusion, this review provides a comprehensive overview of the association between ghrelin and GI disorders. Ghrelin, being the sole hormone responsible for appetite stimulation, has undergone extensive investigation since its initial identification, which has led to the discovery of its multiple functions. Notably, its role in stimulating appetite and promoting gastric motility renders it a significant target in GI disorders. Multiple studies have provided evidence indicating the protective role of ghrelin in the development of esophageal disorders, gastric disorders, GI functional disorders, and CC. Additionally, several clinical trials have demonstrated the effectiveness of ghrelin and its receptor agonists in the management of these GI diseases, with certain treatments currently being investigated in clinical trials. However, there is a lack of consensus regarding the involvement of ghrelin in the pathogenesis of IBD, which necessitates further research to determine the precise impact of ghrelin in this context. Meanwhile, there is growing interest among researchers regarding the potential utility of ghrelin as a biomarker for GI tumors. Nevertheless, the findings have yielded inconsistent results, potentially attributable to factors such as disease stage, nutritional status of the patients, and the presence of underlying comorbidities. Therefore, further research is warranted to adequately stratify or eliminate these confounding variables. Eventually, additional investigations are necessary to comprehensively clarify the precise function of ghrelin system in GI disorders. This would help facilitate the development of efficacious pharmaceutical interventions for the treatment of GI disorders.

## Glossary

GI: Gastrointestinal
IBD: inflammatory bowel disease
CCK: cholecystokinin
GIP: glucose-dependent insulinotropic peptide
GLP-1: glucagon-like peptide-1
PYY: peptide YY
MMCs: migratory motor complexes
GOAT: ghrelin-O-acyltransferase
GHSR: growth hormone secretagogue receptor
PC1/3: prohormone convertase 1/3
MBOAT: membrane-bound O-acyltransferases
HHAT: hedgehog acyltransferase
LEAP2: liver-expressed antimicrobial peptide 2
CNS: central nervous system
NTS: nucleus tractus solitarius
ARC: arcuate nucleus
NPY: neuropeptide Y
AgRP: agouti-related peptide
DVC: dorsal vagal complex
VTA: ventral tegmental area
POMC: peptide pro-opiomelanocortin
GABA: gamma-aminobutyric acid
AP: area postrema
PVN: paraventricular nucleus
GERD: gastroesophageal reflux disease
MCH: melanin-concentrating hormone
*H. pylori*: *Helicobacter pylori*
Bcl-2: B-cell lymphoma 2
Bax: Bcl-2-associated X
LPS: lipopolysaccharide
PI3K: phosphatidylinositol 3-kinase
UC: ulcerative colitis
CD: Crohn’s disease
DSS: dextran sulfate sodium
TNBS: 2,4,6-trinitrobenzene sulfonic acid
FD: functional dyspepsia
IBS: irritable bowel syndrome
IBS-D: diarrhea-predominant IBS
IBS-C: constipation-predominant IBS
CC: cancer cachexia
